# Advances in the pathogenesis of MASLD and its association with neutrophils

**DOI:** 10.3389/fmed.2026.1792366

**Published:** 2026-03-11

**Authors:** Li Lin, Shuangfeng Zi, Guangcong Zhang, Yanan Ma, Ru Mai, Xuemei Jiang

**Affiliations:** 1 Department of Gastroenterology, Hainan Affiliated Hospital of Hainan Medical University (Hainan General Hospital), Haikou, China; 2 Department of Critical Care Medicine, Zhongshan Hospital, Fudan University, Shanghai, China; 3 Department of Gastroenterology and Hepatology, Zhongshan Hospital of Fudan University, Shanghai, China

**Keywords:** immunity, inflammation, metabolism, recruitment, treatment

## Abstract

Metabolic Dysfunction-Associated Steatotic Liver Disease (MASLD), previously known as Nonalcoholic Fatty Liver Disease (NAFLD), is characterized by an abnormal accumulation of fat in the liver. The causes of MASLD are varied, and include an unhealthy lifestyle, hyperlipidemia, hypertension, and type 2 diabetes mellitus. They are considered to be susceptibility-inducing factors for MASLD, and are strongly associated with obesity. Many researchers have explored the pathogenesis of MASLD in recent years, and have concluded that its development is associated with insulin resistance and impaired fat metabolism, which manifests as a form of metabolic liver injury. Currently available treatments remain challenging to implement, and impose a significant burden on the healthcare system. Neutrophils play an important role in the development of MASLD, and are crucial immune cells that provide the first line of defense against infection and injury. This review examines the pathogenesis of MASLD and the role of neutrophils in the disease, and further explores current advances in MASLD treatment as well as the therapeutic potential of neutrophils.

## Introduction

Metabolic Dysfunction-associated Steatotic Liver Disease (MASLD) is a form of metabolic liver injury that is characterized by hepatic steatosis associated with at least one cardiometabolic risk factor and no other identifiable etiology. Previously known as Nonalcoholic Fatty Liver Disease (NAFLD), it was renamed as the “Metabolic Dysfunction-associated Fatty Liver Disease” (MAFLD) in 2020, and was renamed again as MASLD in 2023 (1).

The pathogenesis of MASLD is complex, and it is now generally accepted that the intake of a high-calorie, high-fat diet is one of its major causes. Such an unhealthy diet leads to an excessive accumulation of lipids in the liver, while contributing to an increase in adipose tissue catabolism as well as a rise in hepatic lipogenesis (2). In addition, insulin resistance (IR) and genetic predisposition are strongly implicated in the development of MASLD. Their interactions may lead to serious liver diseases, such as cirrhosis and hepatocellular carcinoma, in severe cases.

The global prevalence of MASLD varies significantly between countries, with Latin America, the Middle East, and North Africa having a significantly higher prevalence than the Asiar diseases, such as cirrhosis and hepatocellular carcinoma, in severe cases of lipogenesis (2). Its incidence continues to rise at present (3). The age-adjusted mortality rate associated with MASLD has increased significantly from 1999 to 2022, with an average annual rate of change of 10% (4). According to the projection by Kan et al., the global prevalence of MASLD is expected to reach 928.10 cases per 1,00,000 population by 2045, corresponding to 667.58 million incident cases and posing a substantial disease burden worldwide (5). Liver-related complications have become the leading cause of death in patients with MASLD in the United States (4). Metabolic-associated Steatohepatitis (MASH) progression to clinically relevant endpoints—such as all-cause mortality, cirrhosis, and need for liver transplantation—is predominantly determined by the degree of fibrosis, and is commonly accompanied by cardiometabolic conditions including type 2 diabetes (T2DM) and hypertension (6). We should thus treat people at high risk of MASLD by using proactive and preventive approaches to minimize the likelihood of the progression of this disease to fibrosis, or even liver cancer.

A meta-analysis revealed that, compared with healthy controls, patients with MASLD exhibited significantly elevated levels of both the Neutrophil Percentage-to-Albumin Ratio (NPAR) and the Neutrophil-to-Albumin Ratio (NAR) (7). This suggests that neutrophils are closely related to the occurrence and development of MASLD, and play a key role in the body's immune system. When an inflammatory response occurs, neutrophils are usually the first cells to be recruited to the area of inflammation (8). In case of liver injury, neutrophils and various other inflammatory cells gather at the injury site through the action of multiple mediators and signaling molecules. Neutrophils function differently under different pathophysiological conditions. They may either contribute to the development of hepatic inflammation through the release of inflammatory mediators and other pathways, or may play an anti-inflammatory role—for example, by releasing certain substances with anti-inflammatory properties, or by participating in immune-regulatory mechanisms to slow down the course of MASLD.

Here, we discuss the pathogenesis of MASLD, and the mechanisms involved in the transition from it to MASH. We focus on analyzing the key role of neutrophils in the development of MASLD.

## The MASLD disease spectrum: from steatosis to MASH, fibrosis, and HCC

While MASLD is a subject of significant research interest worldwide, its precise mechanism has yet to be elucidated. Various non-alcoholic factors, including IR, lipotoxicity, inflammation, and gut microbiota (GM), are considered potential mechanisms of MASLD, which is characterized by functional metabolic disorders, thereby leading to a series of hepatic pathological changes.

IR is currently considered to be the main mechanism underlying MASLD. The process begins with simple steatosis resulting from excessive caloric intake, characterized by substantial accumulation of triglycerides within hepatocytes. In overweight individuals, excessive deposition of diacylglycerol (DAG) activates protein kinase Cε (PKCε), which translocates to the cell membrane to inhibit insulin signaling (9). Hepatic IR reduces intrahepatic glycogen synthesis and promotes gluconeogenesis, thereby leading to hepatic lipid accumulation, although hepatocytes remain intact at this stage. This sequence subsequently triggers hepatic steatosis, marking the onset of MASLD. Lipotoxicity is further triggered under the action of IR and lipid deposition, and can induce pathological changes such as oxidative stress, organelle dysfunction, and apoptosis (10), ultimately leading to the development of MASH. MASH is characterized by inflammation and hepatocellular damage (11) that may progress to this stage in a significant proportion of cases (12).

**Figure 1:**
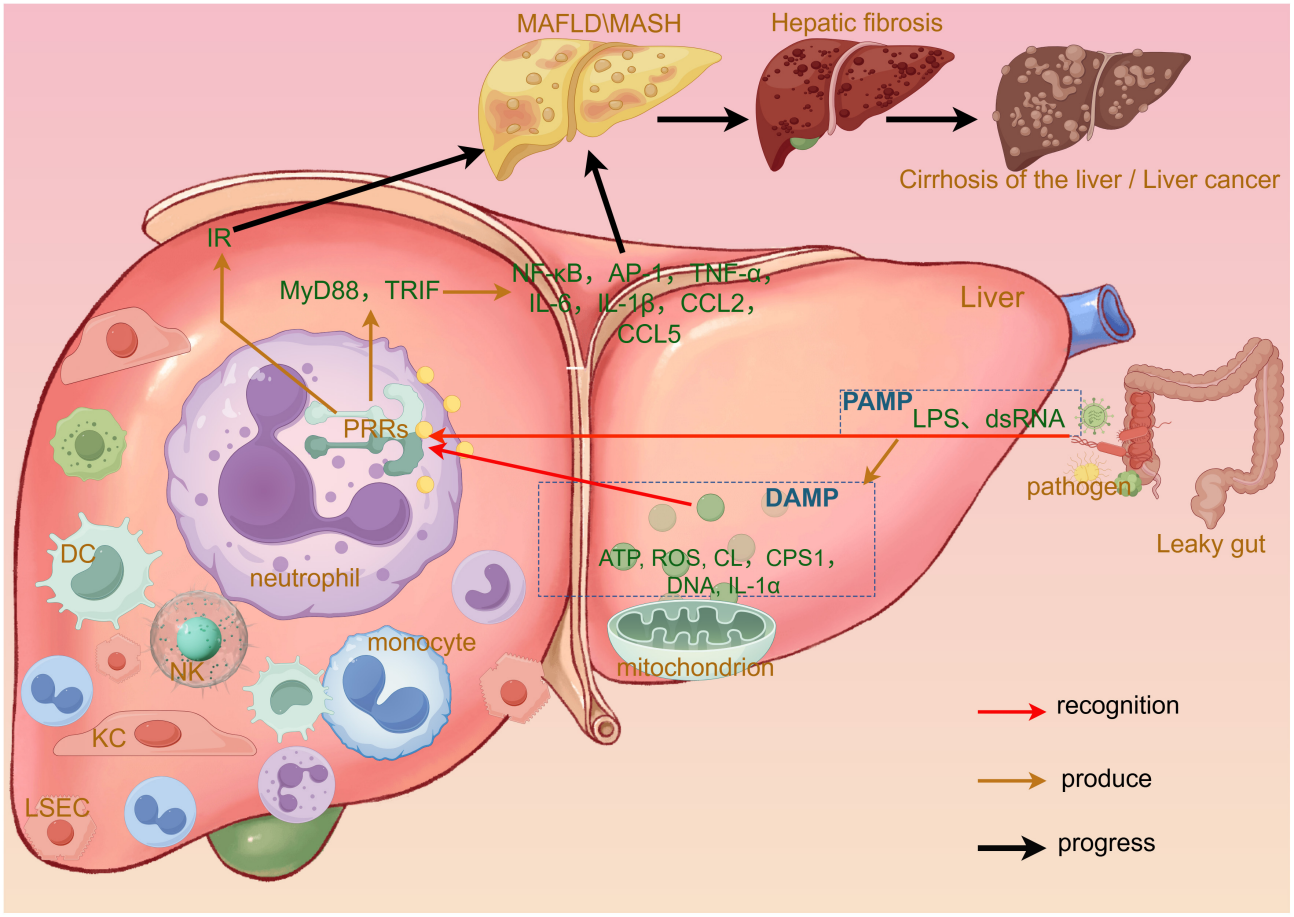
Specific mechanisms by which PAMPs and DAMPs contribute to the development of MASLD. “PAMP” refers to increased hepatic exposure to pathogens due to such conditions as intestinal barrier dysfunction (often described as a “leaky gut”). DAMPs, on the contrary, are the products of sterile inflammatory responses. When the liver is damaged, including in contexts of increased PAMPs, the hepatocyte mitochondria release endogenous biomolecules such as ATP, ROS, CL, CPS1, mitochondrial DNA, and IL-1α, which act as DAMPs. The presence of PAMPs or DAMPs triggers the recruitment of numerous immune cells to the liver, including neutrophils, monocytes, KCs, DCs, NK cells, and LSECs. These PAMPs and DAMPs are recognized by PRRs. On the one hand, this recognition exacerbates hepatic insulin resistance and, on the other, activates such signaling adaptors as MyD88 and TRIF within the liver. This in turn initiates a cascade of inflammatory responses that lead to the production of various inflammatory mediators, including NF-κB, AP-1, and TNF-α. PAMPs, pathogen-associated molecular patterns; DAMPs, damage-associated molecular patterns; IR, insulin resistance; CL, cardiolipin; CPS1, carbamoyl phosphate synthetase 1; DC, dendritic cell; KC, kupffer cell; NK, natural killer; PRRs, pattern recognition receptors; LSEC, liver sinusoidal endothelial cells.

It has been suggested that inflammation in the context of MASLD and MASH is associated with the hepatic immune response, and innate hepatic immunity is induced by molecules from pathogens sensed through specific pattern recognition receptors (PRRs) (13). These are molecules with pathogen-associated molecular patterns (PAMPs) and damage-associated molecular patterns (DAMPs) (Figure 1). PAMPs originate from intestinal dysbiosis and/or intestinal leakage, whereas DAMPs originate from dead/damaged hepatocytes and other cells. DAMP and PAMP molecules are detected by PRRs or inflammatory vesicle sensors in innate immune cells such as neutrophils, which subsequently trigger a series of inflammatory responses and generate a number of pro-inflammatory cytokines (14), which in turn lead to hepatic inflammation and cellular damage.

The GM plays a crucial role in maintaining the health of the organism, and a disruption in its homeostasis has been identified as a potential mechanism of MASLD (1). Research has shown that an imbalance in the GM is associated with a variety of factors, including IR, nutritional status, and endotoxemia, which together act on individuals with genetic susceptibility. This in turn may induce the development of MASLD (15). On the one hand, an imbalance in the intestinal flora increases the permeability of the intestinal barrier, and may lead to excessive intestinal absorption of fatty acids to cause disturbances in lipid metabolism in the body and ultimately lead to hepatic steatosis (16). On the other hand, a homeostatic imbalance in the GM may also cause intestinal damage and expose the liver to bacteria, thereby increasing the risk of liver injury (10). The deterioration of the intestinal microenvironment causes an increase in the proliferation of gram-negative bacilli, which further produce lipopolysaccharide (LPS). The latter is an endotoxin molecule that exacerbates the progression of MASLD, and enters the liver through the portal vein to activate toll-like receptors (TLRs). The latter in turn activate a series of signaling molecules associated with inflammatory cytokines (17), thus promoting the development of MASLD. These mechanisms highlight the importance of maintaining a balanced GM for the prevention of MASLD^**^

Based on the above mechanisms, MASLD gradually progresses to MASH. If left uncontrolled, and under the influence of various complex factors such as germline variations, altered molecular signaling, and the immune microenvironment, it may ultimately lead to cirrhosis and even hepatocellular carcinoma (HCC) (11, 18). In the context of MASLD, these pathological changes are considered a continuous process encompassing multiple hepatic pathologies, ranging from simple steatosis to inflammatory steatohepatitis—the latter characterized by lobular inflammation and hepatocyte ballooning, and possibly accompanied by fibrosis. If the disease continues to progress, it may further deteriorate into cirrhosis and HCC (19). A large meta-analysis reported that the annual pooled incidence of liver-related events, including liver cancer, was 24.28 per 1,000 individuals. Moreover, the incidence of HCC was significantly higher in patients with biopsy-confirmed MASH than in those without MASH (20).

## Neutrophil recruitment

As the body's primary response cells to injury, neutrophils play a complex role in liver injury as a heterogeneous population of immune cells with both anti-inflammatory and pro-inflammatory functions. Maretti-Mira et al., through transcriptomic analysis, revealed that in the early stages of MASH, mature neutrophils predominate in the peripheral blood. However, as the degree of hepatic inflammation and fibrosis worsens, the bone marrow undergoes emergency granulopoiesis, leading to the premature release of a large number of immature neutrophils into the bloodstream. These cells highly express precursor markers, exhibit a pro-inflammatory phenotype, exacerbate liver injury, and promote the progression of fibrosis (21). These findings highlight that the dynamic evolution of the circulating neutrophil profile is a key feature of MASH progression.

**Figure 2:**
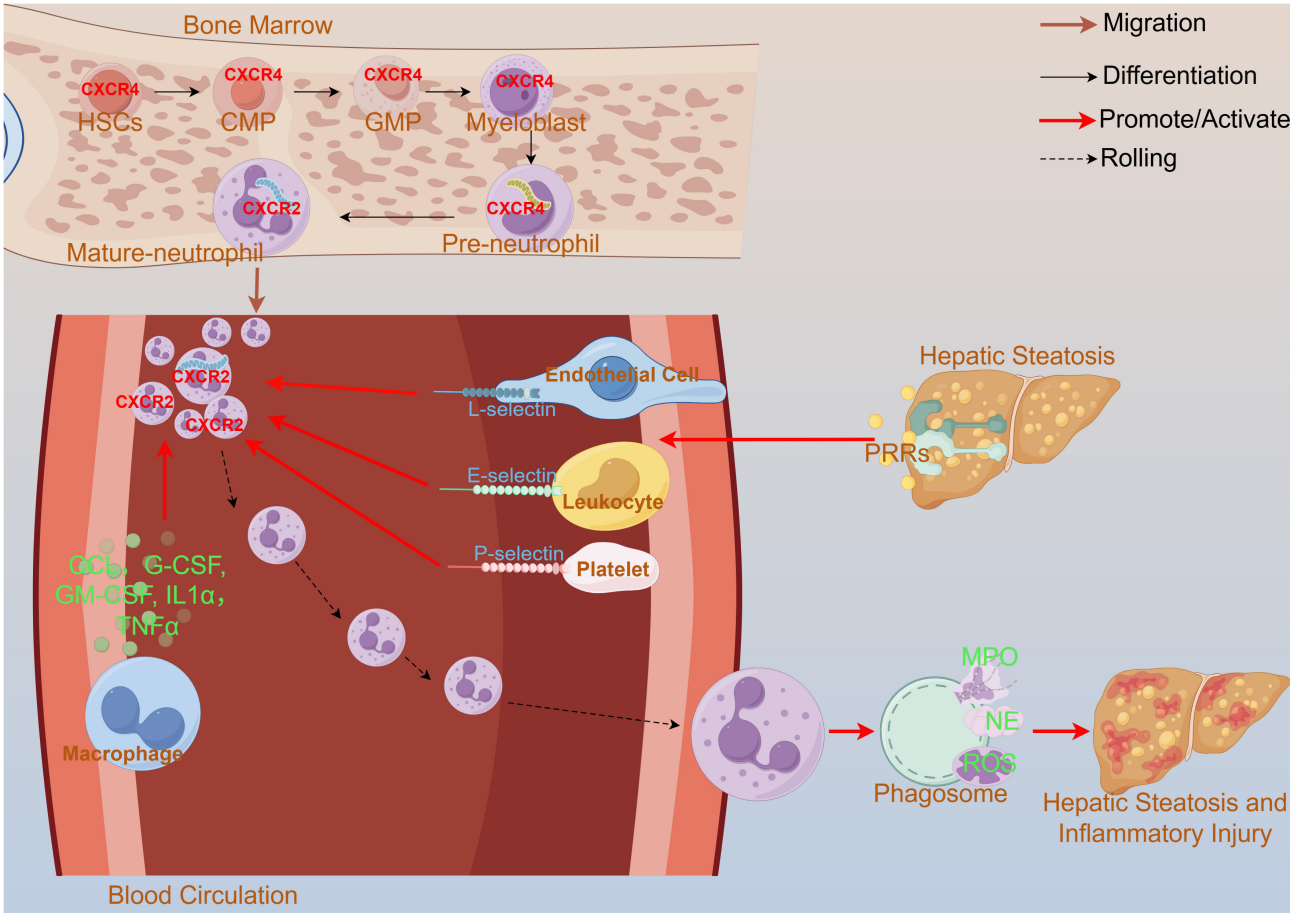
The differentiation, maturation, recruitment, and pro-inflammatory processes of neutrophils. Neutrophils differentiate and mature in the bone marrow, progressing from HSCs through CMPs and GMPs to the myeloblast stage. A high expression of CXCR4 on immature neutrophils causes them to be retained within the bone marrow. Upon maturation, CXCR4 expression decreases while CXCR2 expression increases; CXCR2 promotes the release of neutrophils into the bloodstream to perform their functions. When the liver is damaged, PRRs detect the damage and activate selectins on the platelets, endothelial cells, and leukocytes. The macrophages secrete such factors as CCL, G-CSF, and IL-1α. Through coordinated interactions among these cells, the neutrophils are recruited via a multi-step process involving tethering, rolling, adhesion, crawling, and transmigration. This ultimately leads to their specific accumulation in the liver. Stimulated neutrophils form phagosomes, which initiate cytotoxic responses through the generation of molecules including MPO, NE, and reactive ROS, thereby exerting a spectrum of both anti-inflammatory and pro-inflammatory effects. HSCs, hematopoietic stem cells; CMPs, common myeloid progenitors; GMPs, granulocyte–macrophage progenitors; PRRs, pattern recognition receptors; CCL, C-C motif chemokine ligand; GM-CSF, granulocyte–macrophage colony-stimulating factor; G-CSF, granulocyte colony-stimulating factor; MPO, Myeloperoxidase; NE, neutrophil elastase; ROS, reactive oxygen species.

Neutrophils have a short lifespan, patrol the bloodstream for only one day after maturation (22), and undergo differentiation, maturation, and pro-inflammatory and apoptotic processes in a short period (Figure 2). When bacterial peptides or inflammatory mediators in the blood, such as complement factors, bind to receptors on the surface of neutrophils, the latter are activated and recruited to the damaged areas of the liver. Lauszus et al. showed that the levels of expression of CD62L and CD11b on neutrophils were elevated in the blood of patients with MASH, while CD11b expression was enhanced in MASLD patients. These changes were not observed in patients with other liver diseases, which suggests that neutrophil migration may be associated with the progression of MASH (23). Hwang et al. found that the overexpression of CXCL1 enhanced the expression of chemokines in hepatocytes that recruit neutrophils in a mouse model raised on a high-fat diet (HFD) (24). This upregulated chemokine not only attracts neutrophils, but also promotes the recruitment of lymphocytes that further enhance neutrophil aggregation (25). In addition, CXCL6 has a similar function to that of CXCL1, and promotes neutrophil recruitment by binding to CXCR1 and CXCR2 receptors. The presence of CXCL5 on platelet extracellular vesicles has also been shown to have a positive effect on neutrophil adhesion, as have IL-1α and the C-C motif chemokine ligand CCL2 (26). Research has also shown that adipose tissue macrophages enhance neutrophil recruitment to exacerbate liver injury (27) by releasing a range of pro-inflammatory factors and cytokines, including CCL2, TNF-α, IL-1β, and IL-6, which contribute to neutrophil recruitment at the site of liver injury (28). These findings show that neutrophils are involved in hepatic inflammation, and reveal the factors that play a regulatory role in the initiation of this process as well as the potential mechanisms by which neutrophils are involved in the progression of liver disease. How do neutrophils precisely localize to the site of liver injury? Some studies have revealed interactions between neutrophils and adhesion molecules on endothelial cells, such as E-selectin, that promote the accurate migration of neutrophils to the damaged tissue. E-selectin levels are significantly elevated in MASH patients, a phenomenon that suggests a possible key role for it in disease progression (24). This elevated level of E-selectin may be associated with neutrophil recruitment, which in turn is involved in the process of liver injury and the inflammatory response to it (24).

In addition to the above facilitators, there are factors in the organism that inhibit neutrophil aggregation and recruitment: that is, High-density Lipoprotein Cholesterol (HDL) inhibits neutrophil activation and migration, thus suppressing hepatic inflammation (28). We define the ratio of neutrophils to HDL as the NHR. Studies have shown that the higher the NHR is, the higher is the prevalence of MASLD. This suggests a possible negative correlation between the level of HDL and the prevalence of MASLD.

## Effector mechanisms of neutrophils in MASLD liver injury

### Neutrophil interactions with intrahepatic cells

Neutrophils indirectly influence the progression of MASLD by interacting with multiple cell types. These interactions generate various cytokines, thereby exerting either pro-inflammatory or anti-inflammatory effects on the liver (Table 1).

**Table 1:** Cell types that interact with neutrophils.

**Cell**	**Interaction mechanisms**	**References**
CD4+/CD8+ T cells	Neutrophils suppress the activity of CD4^+^and CD8^+^T cells, thereby compromising the immune defense of liver tissue against injury.	(53)
HSCs	The oxidative stress induced by neutrophils drives the activation of hepatic stellate cells (HSCs).	(54)
Monocyte	NETs activate monocytes and promote the production of inflammatory cytokines by them.	(55)
LSEC	Promotes neutrophil recruitment.	(56)
Platelet	Promotes intrahepatic NET formation.	(57)
KCs	Differentiate into M1 or M2 phenotypes, exerting pro-inflammatory and anti-inflammatory effects in the liver, respectively.	(58)

Neutrophils suppress the proliferation and activation of CD4?/CD8?T cells, thereby reducing the immune defense of the liver tissue against injury, and contributing to the progression from MAFLD to MASH. Moreover, the neutrophils generate NETs, which induce the activation, proliferation, and migration of the HSCs. Meanwhile, the neutrophils produce MPO. MPO-associated lipid peroxidation products can stimulate the stellate cells to synthesize type-I collagen. Furthermore, neutrophil-derived NETs activate the monocytes and promote the production of more inflammatory cytokines by these cells. The mechanical stretching of LSECs upregulates the expression of the neutrophil chemokine CXCL1. Moreover, platelet accumulation in the liver protects against invading microorganisms by promoting local NET formation. KCs can be classified into pro-inflammatory M1 and anti-inflammatory M2 phenotypes. Various factors, including NETs and DAMPs, influence the polarization of KCs toward either the M1 or M2 phenotype, thereby exerting pro-inflammatory or anti-inflammatory effects, respectively. HSCs, hepatic stellate cells; NETs, neutrophil extracellular traps; LSECs, liver sinusoidal endothelial cells; KCs, kupffer cells; DAMPs, damage-associated molecular patterns.

### Effector molecules and functions of neutrophils

#### Involvement of NETs in the process of MASLD

NET structures are formed through the discharge of nuclear material into the extracellular space by neutrophils (24). The interaction between platelets and neutrophils also influences NET formation. Specifically, platelets bind to the bacteria on inflamed surfaces to form platelet–bacterial bundles that promote the formation and release of NETs (26). The formation of NETs is also associated with the NETosis process, which is a complex meshwork of extruded nuclear or mitochondrial DNA and various inflammatory mediators in a protease scaffold (29). These fibers are modified by anti-microbial enzymes and histones that have the ability to trap and destroy microorganisms, thus limiting their spread (30). Although NETs can kill bacteria, the formation of excess NETs can lead to worsening inflammation in case of MASH (29). Moreover, they can cause tissue damage if their regulation is unbalanced. NETs are formed during the early stages of MASLD through the signaling of the S1P receptor 2, which has a pro-inflammatory effect. NETs also attract neutrophils to infiltrate the liver and promote neutrophil–monocyte/macrophage interactions in MASH (30) to accelerate inflammation.

#### Reactive oxygen species (ROS)

**Figure 3:**
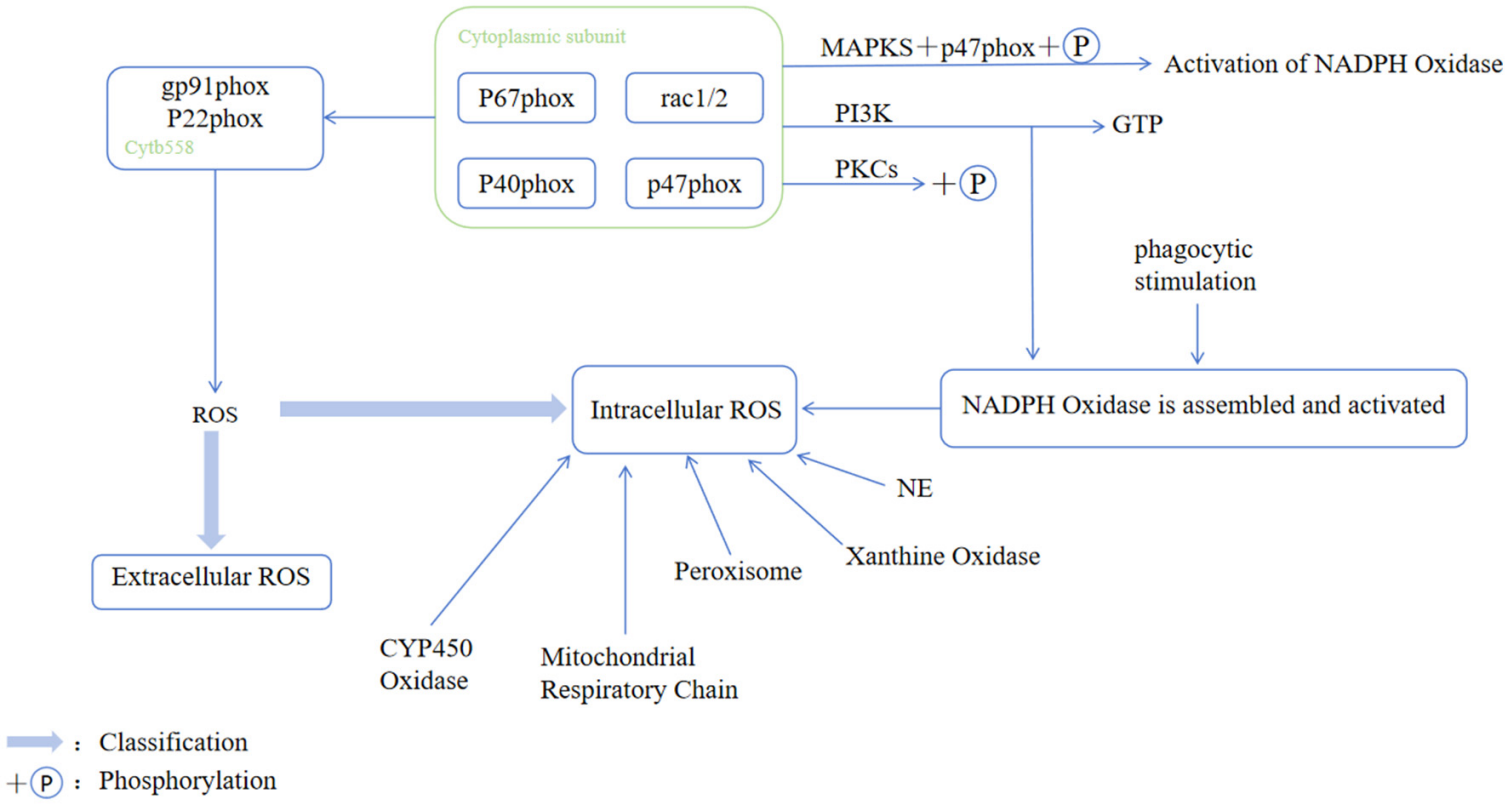
Activation of the NADPH oxidase complex and pathway for ROS generation in phagocytes. Upon external stimulation, signaling pathways such as PKC and PI3K are initially activated, and this leads to the key phosphorylation of the cytoplasmic subunit p47phox via downstream MAPKs. This phosphorylation facilitates the formation of an activated complex in the cytoplasm comprising p47phox, p67phox, p40phox, and GTP-bound Rac1/2, where this subsequently translocates to the cell membrane. The complex then assembles with the membrane-bound cytochrome b558 (composed of gp91phox and p22phox) to form the active NADPH oxidase. This enzyme utilizes NADPH as an electron donor to catalyze the reduction of oxygen, thus generating a superoxide anion. While its primary product is an extracellular ROS, it also contributes to intracellular ROS levels. Furthermore, the diagram shows other intracellular sources of the baseline ROS, including the mitochondrial respiratory chain, peroxisomes, xanthine oxidase, cytochrome P450 oxidases, NADPH oxidase, and neutrophil elastase. CYP450, ytochrome P450; NE, neutrophil elastase; NOX, NADPH oxidase; PKC, protein kinase C; PI3K, phosphatidylinositol 3-kinase.

Both NETs and two major enzyme families—NADPH oxidase and CYP450—release the ROS during the development of MASH (15, 31) (Figure 3). Neutrophils are also important sites for ROS production, and produce much higher levels of ROS than nonphagocytic cells. Low levels of ROS production rely mainly on mitochondrial respiration (32). The ROS are considered to be a key factor in promoting liver fibrosis (15), which exacerbates oxidative stress in the liver. Specifically, overfeeding leads to the production of excess fatty acid from excessive lipid aggregation, which in turn leads to a high β-oxidation rate. This increases the production of the ROS, which exert a pro-inflammatory effect by disrupting the structure and function of the membrane (33). It also contributes to the progression of MASH through the activation of the hepatic stress kinase Apoptosis Signaling-regulated Kinase 1 (ASK1) and p38 (26). In addition, neutrophils and the ROS secreted by them can directly activate hepatic stellate cells, which may exacerbate the pathological process of MASLD. Furthermore, these mechanisms together constitute a complex network of roles of neutrophils in inflammatory and infectious responses (34).

#### Myeloperoxidase (MPO)

MPO is mainly released by Polymorphonuclear Neutrophils (PMNs), and is the main protein secreted by neutrophils upon activation (35). In addition, KCs and macrophages secrete small amounts of MPO, which is a major driver of lipid peroxidation in inflammatory tissues, and is expressed by KCs in the fibrotic septa in case of cirrhosis. Koop et al. found that hepatocyte injury was attenuated in MPO-deficient mice, suggesting a correlation between MPO and the development of MASLD (36). MPO exhibits chlorinating activity that catalyzes the conversion of hydrogen peroxide and chloride ions into hypochlorous acid—a function that induces oxidative stress to promote MASH (35). Neutrophil MPO also triggers HSCs and promotes liver fibrosis (27). On the contrary, MPO, a signature enzyme of neutrophils, also has the ability to kill pathogens, and is a key component in the anti-inflammatory response of neutrophils (25).

#### Neutrophil serine proteases (NSPs)

NSPs are key effector molecules that are stored as active enzymes within neutrophil granules, and enable a rapid response upon stimulation. They comprise several distinct subtypes. Their full activation is regulated by Dipeptidyl Peptidase I (DPPI), which cleaves the N-terminal dipeptides in the endoplasmic reticulum and the Golgi apparatus to ensure timely protease maturation (37). Upon neutrophil activation, the released NSPs play a dual role in inflammation. In the extracellular milieu, they function as pro-inflammatory mediators by processing and activating a broad spectrum of cytokines and chemokines to amplify the inflammatory cascade (25). Concurrently, certain NSPs can enter the cytoplasm of the target cells and modulate signaling pathways, where this contributes to anti-inflammatory responses. Furthermore, NSPs possess direct anti-microbial properties: They can kill bacteria and hydrolyze the host proteins to generate anti-microbial peptides, and can deactivate virulence factors that are essential for bacterial pathogenesis (37). This multi-faceted functionality allows NSPs to play a complex, yet critical, role in regulating inflammatory processes (25). Given these mechanisms, artificially localizing or modulating NSP activity within the liver cells presents a promising therapeutic strategy for MASLD.

#### Lipid carrier protein 2 (LCN2)

LCN2 is a circulating protein present in the azurophilic granules of neutrophils. Its presence is important for controlling intrahepatic lipid homeostasis and inflammation (38). LCN2 can increase the pool of matrix metalloproteinase 9 in human neutrophil granulocytes, and is secreted by prepackaged neutrophil granules at the onset of inflammation. These granules participate in the anti-inflammatory process by restricting bacterial growth, thus inhibiting liver inflammation in patients with MASLD (39). In addition, LCN2 helps regulate lipid metabolism and IR in the body (39). It also up-regulates the expression of Periostin 5 (PLIN5/OXPAT), which maintains the balance between lipogenesis and catabolism to regulate lipid metabolism (40) and exert an inhibitory effect on MASLD. At the same time, LCN2 increases IR (39), which implies that it aggravates MASLD IR.

#### Calreticulin

Calprotectin is a calcium- and zinc-bound protein dimer that is abundant in the cytoplasm of neutrophils. It may be involved in the formation of inflammatory responses in case of MASLD (41). Bourgonje et al. demonstrated that plasma calprotectin was significantly elevated in patients with suspected MASLD, even after adjusting for identified risk factors for it (41). However, our current understanding of the relationship between calprotectin and MASLD is limited, and there is considerable room for further research on the role of calprotectin in promoting the development of liver fibrosis and cirrhosis, and even its progression to HCC in patients with MASLD.

## Pharmacological treatment of MASLD

### Treatment strategies for patients with simple MASLD

MASLD refers to hepatic steatosis in the presence of one or more cardio-metabolic risk factors, such as T2DM or moderate hyperglycemia, in the absence of harmful alcohol consumption (42). By definition, the pathogenesis of MASLD is closely linked to metabolic dysregulation, and this underscores the need for a multi-faceted therapeutic approach. Although pharmacologic interventions are still evolving, several strategies can be implemented to modulate disease progression. One actively studied treatment strategy involves directly targeting the hepatocytes, including by using Thyroid Hormone Receptor β (THRβ) agonists and Fatty Acid Synthase (FAS) inhibitors. Notably, resmetirom—a THRβ agonist—was recently approved by the U.S. FDA as the first drug specifically indicated for MASH (43). Resmetirom is a highly selective THRβ agonist that acts specifically on hepatocytes. By selectively activating hepatic THRβ, it enhances mitochondrial fatty acid β oxidation and reduces hepatic lipid accumulation, thereby improving lipid metabolism, suppressing inflammatory pathways, and promoting anti-fibrotic remodeling with generally mild and infrequent adverse effects (44).

### Treatment strategies for patients with comorbidities

The International Diabetes Federation (IDF) has recently proposed a systematic, stratified diagnosis and treatment strategy, along with targeted treatment recommendations for patients with comorbid MASLD and T2DM (42). These recommendations include a variety of approved therapeutic approaches for managing the comorbidity. This suggests that an integrated co-management model for T2DM and MASLD will become essential in the future. Research is currently underway on pharmacological interventions that simultaneously target both conditions. Table 2 provides a summary of the latest advances in research on drug therapy for this comorbidity.

**Table 2:** Pharmacological treatment of MASLD.

**Category**	**Drug**	**function**	**References**
Anti-hyperglycemic drugs	Pioglitazone	Significantly improve liver histological properties and insulin sensitivity	(59)
	SGLT2	Significantly reduce VAT, SAT, and ectopic liver fat	(60, 61)
	Metformin	Reduce serum lipid and glucose levels	(62)
	GLP-1 RA	Mitigate liver damage and metabolic disorders in patients with MASLD	(63)
Fc–FGF21 analog	Efruxifermin	Combination therapy with GLP-1 RA has been shown to significantly reduce hepatic fat content, ameliorate liver injury and fibrosis, and improve insulin resistance	(64)
Pan-PPAR agonist	Lanifibranor	Significantly improves IR in the liver, muscle, and adipose tissue	(65)
VitE	/	Significantly improves liver inflammation and histological serum markers in patients with MASLD	(66)

SGLT2, sodium–glucose cotransporter 2; VAT, visceral adipose tissue; SAT, subcutaneous adipose tissue; DPP-4, dipeptidyl-peptidase-4; GLP-1 RA, peptide-1 receptor agonist; Fc–FGF21, fragment crystallizable–fibroblast growth factor 21; Pan-PPAR, pan-peroxisome proliferator-activated receptor-α*γδ*; IR, insulin resistance; VitE, vitamin E.

### MASLD subtype-directed therapeutic strategies

Raverdy et al., through data-driven cluster analysis, identified two high-risk MASLD phenotypes: the liver-specific type and the cardiometabolic type. They found that these two phenotypes differ in their underlying pathophysiological mechanisms, clinical trajectories, and therapeutic strategies. In patients with the liver-specific phenotype, the pathology is predominantly confined to the liver; therefore, liver-targeted drugs may offer greater advantages by effectively reducing hepatic fat content and inflammation. For patients with the cardiometabolic phenotype, the primary drivers are systemic metabolic disturbances; thus, priority should be given to medications that ameliorate systemic metabolism and promote weight loss. These agents not only improve hepatic steatosis and inflammation but also significantly reduce the risk of cardiovascular events and type 2 diabetes. Furthermore, patients with the cardiometabolic phenotype may derive greater benefit from lifestyle interventions and intensified management of cardiovascular risk factors (45).

### Neutrophil-targeted therapies: rationale and translational outlook

Current mainstream treatments, such as lifestyle interventions, insulin sensitizers, vitamin E, and GLP-1 receptor agonists, primarily target systemic metabolic disturbances. Although necessary, these approaches often have a slow onset, and demonstrate limited efficacy in patients who have already progressed to significant inflammation and fibrosis. Compared with currently available MASLD therapies that focus mainly on metabolic dysregulation, strategies targeting neutrophil-related mechanisms offer several potential advantages. First, they directly address the execution phase of hepatic inflammation and fibrosis, thus potentially providing alternative interventions for patients who respond poorly to metabolic treatments, or have already entered a state of pronounced inflammation. Second, by targeting localized neutrophil activation or specific mediators within the liver, it may be possible to achieve more precise intrahepatic anti-inflammatory effects while preserving systemic immune defense functions. Furthermore, neutrophil-related biomarkers may be used in the future to identify high-risk progressive phenotypes, where this can enable stratified and precision medicine. Such strategies are not intended to replace traditional metabolic interventions, but rather hold promise for combination therapies. They can help form a multi-target synergistic approach to treatment—particularly during the critical window for preventing the rapid progression of MASH to fibrosis. Future clinical translation should focus on validating liver-targeted strategies for delivery, optimizing biomarkers for patient selection, and rigorously evaluating liver-specific benefits vs. systemic safety in early-phase clinical trials.

By building on the mechanisms of neutrophil involvement, the targeting of neutrophil-derived inflammatory pathways represents a promising translational avenue. Few studies have explored improving hepatic steatosis by modulating neutrophil activity. A major challenge in this regard is that neutrophils, as crucial immune effector cells, play an essential role in anti-microbial defense. Simply suppressing neutrophils to ameliorate MASLD progression can significantly compromise host immune function—a therapeutic strategy akin to “winning the battle but losing the war.” However, if specific markers identifying liver-localized neutrophils that drive MASLD can be precisely identified, it may be possible to confine the pathological processes of liver inflammation and fibrosis to specific genes, inflammatory factors, or metabolic pathways. This approach could enable the development of targeted interventions that act selectively within the liver and precisely against these key factors, thereby minimizing systemic immune disruption. Such a strategy holds promise as a potentially effective and focused therapeutic avenue. Research has demonstrated that the inhibition of specific neutrophil effector functions can ameliorate steatohepatitis and fibrosis in MASLD models. For instance, the use of anti-oxidants to scavenge mitochondrial and cytoplasmic reactive ROS has been shown to prevent simple steatosis and progression to NASH (46). Likewise, NE inhibition or genetic knockout improve inflammatory responses in the adipose tissue and liver under HFD conditions by modulating hepatic insulin sensitivity and signaling in mice (47). Moreover, the suppression of NETosis with a PAD4 inhibitor reduces weight gain in experimental models (48). However, early-phase clinical trials of MPO inhibitors suggest that while such approaches may be tolerable, their efficacy in case of MASLD remains to be evaluated.

Identifying reliable biomarkers of neutrophil-driven liver damage is crucial to enable such targeted interventions as those mentioned above. Candidate markers include the Neutrophil-to-Lymphocyte Ratio (NLR), circulating MPO-DNA complexes as surrogates for NET formation, and the neutrophil-to-high-density-lipoprotein–cholesterol ratio. While these markers have shown associative value in cohort studies, their analytical standardization, longitudinal clinical validity, and specificity for MASLD progression require further rigorous assessment. Future therapeutic strategies should thus aim to selectively inhibit liver-localized neutrophil activation or NET release while preserving systemic anti-microbial immunity—a balance that is essential for translating neutrophil-targeted approaches into viable clinical options.

## Discussion

Taken together, the above shows that neutrophils are recruited to the site of liver injury, and subsequently produce a variety of signaling molecules, including cytokines, chemokines, proteases, NETs, and ROS. All of them have potentially negative effects on MASLD patients. These findings emphasize the role of neutrophils in the pathogenesis of MASLD, and the importance of controlling their activity in therapeutic strategies (34).

Beyond the liver, MASLD is closely intertwined with cardiovascular disease, driven by shared risk factors such as obesity, hypertension, and dyslipidemia, as well as overlapping genetic susceptibility (49–52). The coexistence of MASLD and T2DM further amplifies the risk of major adverse cardiovascular events (MACEs), including myocardial infarction and stroke. Studies have shown that, compared with patients without MASLD, individuals with both MASLD and T2DM face a 1.5–2folds increased risk of myocardial infarction, stroke, and other cardiovascular events (50). These observations underscore the need for an integrated, multi-disease management approach in MASLD patients, particularly those with cardiometabolic comorbidities. Such a strategy holds greater prognostic value than liver-focused interventions alone. However, it requires multidisciplinary and multi-sectoral collaboration; further research is needed to evaluate the feasibility of specific therapeutic measures for patients with diverse comorbidities and to develop individualized management plans. Its clinical implementation therefore remains to be further explored.

Prevalent research in the area suffers from several limitations, including the variability between animal models and human pathological states, and challenges posed by the diversity of the research instruments used. These limitations may affect the generalizability and precision of the results of research. Further in-depth studies on the precise mechanisms of neutrophil action in MASLD are needed to develop more effective therapeutic strategies for it. Neutrophils play an important role in the onset and development of metabolic fatty liver disease, and a sound understanding of their mechanisms provides potential targets for the development of new diagnostic and therapeutic approaches. However, more research is needed to refine our understanding of this complex process to more effectively manage this disease.
